# Intra and Inter-specific Variability of Salt Tolerance Mechanisms in *Diospyros* Genus

**DOI:** 10.3389/fpls.2020.01132

**Published:** 2020-08-07

**Authors:** Francisco Gil-Muñoz, Juan Gabriel Pérez-Pérez, Ana Quiñones, María del Mar Naval, María Luisa Badenes

**Affiliations:** ^1^ Centro de Citricultura y Producción Vegetal, Instituto Valenciano de Investigaciones Agrarias (IVIA), Valencia, Spain; ^2^ Centro de Investigación en Desarrollo de Agricultura Sostenible, Instituto Valenciano de Investigaciones Agrarias (IVIA), Valencia, Spain

**Keywords:** *Diospyros*, saline tolerance, rootstock breeding, persimmon, *HKT-1* gene, salt stress

## Abstract

Saline stress is one of most important problems that agriculture must face in the context of climate change. In the Mediterranean basin, one of the regions most affected, persimmon production can be compromised by this effect, due to the limited availability of salt tolerant rootstocks. Seedlings coming from four populations from the *Diospyros* genus have been exposed to salt stress in order to identify salt tolerance genotypes within these populations. Morphological, physiological, and transcriptomic approaches have revealed different mechanisms of tolerance among the population studied. An *HKT1-like* gene has been shown to have different root expression related to the salt tolerance phenotypes among and within populations. Additionally, we have observed differences in salt-responsive expression among *PIP* aquaporin genes. Genetic variability for salt tolerance can be generated in Diospyros species through crossings and used for overcome salt stress. Furthermore, differences in water use efficiency (WUE) have been obtained between and within populations. The information gathered at transcriptomic and physiological level demonstrated natural and heritable variability among *Diospyros* genus which is the key for salt-tolerant rootstock breeding programs.

## Introduction

Persimmon (*Diospyros* *kaki*) production in the Mediterranean has increased by five times in the last 20 years (Perucho, 2018). In fact, it became an important alternative to citrus production and shares the same crop areas. However, in the recent years, cultural practices and climate change have modified rainfall distribution and have increased salt content in the irrigation water (Visconti et al., 2015). In general, there are two major ways to tackle soil salinization problem, irrigation-and-drainage approach and improvement of salinity tolerance in crops. Saline soils are difficult to remediate, for several reasons that have been outlined by Arzani and Ashraf (2016), and thus the use of salinity tolerant varieties is the most effective and widely used approach. In tree crops composed by rootstock and scion, the best approach to overcome soil salinity is to use salt-tolerant rootstocks compatible with the scion (Forner-Giner and Ancillo, 2013). There are three species of *Diospyros* genus used as rootstocks: *D. kaki*, *D. lotus*, and *D. virginiana* (Badenes et al., 2015). *D. lotus* is the most commonly used rootstock in the Mediterranean area due to its tolerance to lime-filled soils, adaptability, and absence of basal shoots. However, it is high sensitive to salinity (de Paz et al., 2016a; Visconti et al., 2017). *D. virginiana*, is tolerant to salinity and has a good development in lime-filled soils, but it confers too much vigor to the scion and produces basal shoots, both of which are traits that make crop management difficult (Incesu et al., 2014; de Paz et al., 2016b). *D. kaki* is the most used rootstock species around the world, because its compatibility with all the cultivars. This rootstock is not used in the Mediterranean basin because its sensitivity to lime-filled soils and the weak root system developed in those soils that causes pour and slow development (Bellini and Giordani, 2002).

An additional problem for persimmon crop is the origin of the rootstocks; all of them come from seedlings, never from clonal propagation, which implies a high genetic variability among rootstocks. No clonal rootstocks nor clones selected from breeding programs are available, although micropropagation methods have been developed for some genotypes of *D. lotus* and *D. virginiana* (Giordani et al., 2012). Regarding to saline-stress performance, *D. virginiana* has been identified as the most salt-tolerant species and *D. lotus* the most sensitive, being *D. kaki* intermediate (de Paz et al., 2016b; Visconti et al., 2017; Gil-Muñoz et al., 2018a). However, information about the salt tolerance diversity into each rootstock species is not available. To unravel this question is the key for developing genotypes tolerant to salinity. Moreover, to determine the salt tolerance mechanisms among the persimmon rootstock species would allow to define strategies for managing the crop in saline environments and to design interspecific crosses that combined different tolerant mechanisms.

Salt stress tolerance is a polygenic (quantitative) trait highly influenced by environment and genotype-by-environment interaction (Arzani and Ashraf, 2016) (Tiwari et al., 2016). Therefore, discovering the salinity tolerance mechanism or even the genes underlying to the tolerant phenotypes might help to avoid the environment effect in the first steps of a breeding program. Salt stress causes osmotic stress and ion toxicity, leading to cell dehydration, disruption of metabolic processes, nutrient imbalances, membrane dysfunction, and oxidative stress (Essah et al., 2003) (Essah et al., 2003; Arzani, 2008; Munns and Tester, 2008).

Under salinity conditions, osmotic stress causes drought-like effects in the plant. It causes changes in the stomatal aperture of the plant *via* abscisic acid (ABA) route (Fricke et al., 2006). Independently to the water ion profile, this mechanism is activated and might be a drought response effect, as it has been observed that it is regulated by root signals that are also triggered in drought conditions (Termaat et al., 1985; Davies et al., 2005). Photosynthesis of the plant can be severely affected by salinity stress, but the causes are not yet fully known (Gil-Muñoz et al., 2020). Some studies have related salinity stress with an increase in stored carbohydrate (Munns et al., 2000), indicating a reduction in sink demand that may downregulate photosynthesis.

Regulation of water transport, small solutes, and hydric balance through the plant is a mechanism to overcome salinity stress. Maurel et al. (2008) described membrane intrinsic proteins named aquaporins that play a central role in water relations of roots, leaves, and seeds, hence, involved in this regulation. Several studies described changes in aquaporin Plasma membrane intrinsic protein (PIPs) family activity under salt stress (Martínez-Ballesta et al., 2003; Boursiac et al., 2005; López-Pérez et al., 2009) linked to a reduction in hydraulic conductivity (Joly, 1989; Zekri and Parsons, 1989). Furthermore, in citrus rootstocks, it has been observed different aquaporin activity in salt-tolerant genotypes compared to sensitive (Rodríguez-Gamir et al., 2012). Furthermore, experiments on yeast and *Xenopus oocytes* have shown increased Na^+^ conductance when overexpressing *AtPIP2;1* from *Arabidopsis thaliana*, suggesting a possible function for Na^+^ influx into the plant (Chaumont and Tyerman, 2014).

Several genes have been identified with salt tolerance, mainly with ionic stress overcame by avoiding toxic ion inflow, transport, or by balancing ion homeostasis (Arzani and Ashraf, 2016). The Salt Overly Sensitive (SOS) signaling pathway is responsible of maintaining ion homeostasis during salt stress (Zhu et al., 1998). In this pathway, myristoylated calcium-binding protein CBL4 (SOS3) acts as a sensor to perceive the Na^+^-mediated Ca^2+^ spike. After activation, it interacts with a serine/threonine protein kinase (SOS2) (Liu and Zhu, 1998; Halfter, 2000; Liu, 2000; Hrabak et al., 2003) that mediates the activation of the target gene *SOS1* (Shi et al., 2000; Qiu et al., 2002; Quintero et al., 2002; Quan et al., 2007; Quintero et al., 2011), a plasma membrane encoding gene with Na^+^/H^+^ antiporter activity (Qiu et al., 2003) that mediates the retrieval of Na^+^ from the cytosol. However, this pathway has a high demand of energy and can compromise plant growth (Isayenkov and Maathuis, 2019). Other genes related with salt stress tolerance participate in different tolerance mechanisms. Some members of the HKT (High affinity potassium transporter) family have been linked with Na^+^ exclusion (Byrt et al., 2007; Munns and Tester, 2008; Almeida et al., 2013; Isayenkov and Maathuis, 2019) showing a higher expression in roots than shoots (Arabbeigi et al., 2018). Also, a member from the Na^+^/H^+^ exchanger family (NHX) has been liked to salinity tolerance (Apse et al., 1999), possibly *via* a role in stomatal regulation (Barragán et al., 2012). Furthermore, the ALMT (Aluminum-activated Malate Transporter) protein family encodes anion transmembrane channels (Barbier-Brygoo et al., 2011) that have been proposed to have a role in Cl^-^ and Na^+^ sequestration in vacuoles of salt tolerant plants (Baetz et al., 2016).

Plants have developed extensive Na^+^ tolerance mechanisms. Therefore, in saline environments Cl^−^ can become potentially more harmful than Na^+^, as Cl^−^ influx is believed to be mediated by passive mechanisms through anion channels regulated by ABA (Munns and Tester, 2008).

In a previous study, variability of salt tolerance in two *Diospyros* genus species was exploited and studied through interspecific crossings (Gil-Muñoz et al., 2020). However, despite prior works that studied salt stress effect in several *Diospyros* species (Mickelbart and Marler, 1998; Incesu et al., 2014), the mechanisms that regulate the osmotic and ionic stress tolerance within the species used as rootstock in persimmon remain not fully known. Understanding and identifying the mechanisms of salt tolerance present in the natural variability of these species will help to advance in rootstock breeding programs. Screening of physiological parameters and genetic expression of genes involved in tolerance is the strategy proposed for selecting genetic-dependent salt tolerant individuals. Results will provide insights of the mechanisms of tolerance in the persimmon species used as rootstocks. The discovery of the mechanisms for salt tolerance will provide tools for breeding *Diospyros* rootstocks better adapted to the saline conditions present in the Mediterranean basin environment.

## Materials and Methods

### Plant Material and Salinity Treatment

Four populations were used for the salinity tolerance study: Seedlings from an open pollination family of *D. kaki* (DK), seedlings from open pollination family of *D. virginiana* (DV), seedlings from a half-sibling backcross family between *D. kaki x D. virginiana* (BC) and a population from *D. lotus* (DL), a full sibling family from a cross between two *D. lotus* trees at the IVIA persimmon germplasm bank was added to the study (**Supplementary Figure 1**). At the end of March, seeds were stratified for 30 days in plastic bags filled with perlite in a cold chamber at 4°C. After stratification, seeds were transferred to peat-moss and perlite (4:1 ratio, respectively) and kept in a greenhouse at 18–24°C during 2 months (from April 24 to June 26, 2017).

One hundred and twenty-seven seedlings of each population were transplanted into 1-L pots containing coarse sand to ensure complete drain. The plants were distributed randomly in the greenhouse and watered with a nutrient solution (3% Cristaljisa 18-18-18, soluble fertilizer with micronutrients) until apical meristem growth was observed. The plants were acclimated before exposition to salinity treatment, meaning that they were watered with the nutrient solution until normal growth was observed. After acclimation, plants were submitted to a salinity treatment during 60 days. The treatment consisted in 40 mM NaCl added to the nutrient solution. All the watering of the plants was done three times a day with exclusively nutrient solution with 40 mM NaCl until water leakage to ensure soil washing between waterings. Although this salt concentration may appear to be mild, it was chosen based on our previous findings in persimmon (Gil-Muñoz et al., 2018a). The control plants remained watered with the standard nutrient solution.

### Agro-Morphological Data

The parameters total height (cm), leaves (no.), nodes (no.), internodes (cm), and defoliation (1-no. leaves/no. nodes) were measured at days 0, 30, and 45 for all plants (127 of each population). Twenty plants (10 tolerant and 10 sensitive) of each family were selected based on salt tolerance according to the visual and agro-morphological data for further analyses. For the *D.* *lotus* family only five plants could be selected as tolerant. Ten control plants of each population were randomly selected. At the end of the salinity treatment (day 60), prior parameters were also measured for selected plants. Variables related to growth were calculated as the ratio between initial and final value. Relative growth rate (RGR) was calculated as follows:

$$\text{RGR}=\frac{Ln(Height\;2-Height\;1)}{t2-t1}$$

Based on visual symptoms, salinity injury was rated from 0 to 4: 0, no symptoms; 1, leaf turgor loss; 2, leaf tip necrosis; 3, leaf margin necrosis; 4, defoliated plant (**Supplementary Figure 2**).

### Stem Water Potential

Stem water potential **(**ψ_H_, MPa) was measured in three fully expanded leaves of each plant from control and selected plants in a sunny day using a Model 600 Schölander Pressure Chamber (PMS Instrument Company, Albany, OR, USA) at the end of the salinity treatment (day 60). Previously, the leaves was kept in a reflective plastic bag for 30 min to remove water loss (Levin, 2019).

### Leaf Gas Exchange Parameters

Leaf net CO_2_ assimilation rate (A_CO2_), stomatal conductance (g_s_), leaf transpiration rate (E), and internal CO_2_ concentration (C_i_) were measured on single attached leaves from selected (tolerant, sensitive, and control) plants. Determinations were performed using three uniform fully expanded leaves from the mid-stem zone. Intrinsic leaf water use efficiency (WUE) was calculated as A_CO2_ and g_s_ ratio. All measurements were carried out in a sunny day between 9:30 a.m. and 12:30 p.m. at the end of the salt treatment (day 60). Photosynthetically active radiation (PAR) at the leaf surface was adjusted to a photon flux density of 1.000 µmol m^-2^ s^-1^. A CIRAS-2 Portable Photosynthesis System (PP Systems, Amesbury, Massachusetts, USA) was used for the measurements. Leaf laminae were fully enclosed within a PLC 6 (U) universal leaf autocuvette in a closed-circuit model and kept at 25 ± 0.5°C, with a leaf-to-air vapor deficit of about 1.7 kPa. The air flow rate through the cuvette was 0.5–1.5 L min^-1^.

### Ion Content Analysis

After saline treatment, three leaves from each selected plant (control, tolerant, and sensitive plants) were collected. Sample pre-treatments for ion content measurement were performed as described in Gil-Muñoz et al. (2018b). Chloride concentration (mg ml^-1^) was determined by silver ion-titration (Gilliam, 1971) with a Corning 926 automatic chloridometer (Corning Ltd. Halstead Essex, UK). Na^+^, Ca^2+^, K^+^, Mg^2+^, P, and S ions were quantified (mg g^-1^ dry w.t.) using a multiple-collector inductively coupled plasma mass spectrometry (MC-ICP MS, Thermo Finnigan Neptune).

### Gene Expression Analysis

Young fully expanded leaves and root tip tissue was collected after 60 days of salt treatment and immediately frozen and powdered using liquid nitrogen. Control samples from all populations were also collected and processed. Three biological replicates were collected for each individual. RNA was isolated according to Gambino et al. (2008). DNA was removed with the RNase-Free DNase Set (Qiagen, Valencia, CA, USA), using the RNeasy Plant Mini Kit (Qiagen). Purified RNA (500 ng) was reverse transcribed with PrimeScript RT Reagent Kit (Takara Bio, Otsu, Japan) in a total volume of 10 μl.

Six putative orthologous genes involved in different mechanisms leading to salt tolerance were analyzed *SOS1*, *SOS2*, *SOS3*, *NHX1*, *HKT1*, and *ALMT9* with primers designed from *D. lotus* SRA archive (SRA ID: SRP045872) *cv*. Kunsenshi (Akagi et al., 2014). For the *PIP* aquaporin expression analysis, *Arabidopsis thaliana* PIP genes from the PIP1 and PIP2 families were blasted against the *D. lotus* (SRA ID: SRP045872) *cv*. Kunsenshi (Akagi et al., 2014). The output fragments were manually assembled to complete putative orthologous genes. Specific persimmon primers were designed using the sequences obtained (**Table 1**). For PIP expression analysis, individual biological replicates were mixed into three global biological replicates for each population subset.

**Table 1:** Primers used for RT-qPCR analysis.

Gene name	Sequence (5’-3’)
SOS1-Like	F: GGATTTTCTCTGGAAGGAAAGTGCTA R: GGAGATGTAATCAGTTCCTCTTTGACAC
SOS2-Like	F: TTAGAGTTTGTTACTGGAGGGGAACT R: CACTCAGTCCAAAGTCAGAAACCTTCA
SOS3-Like	F: GAAGTTGAGGCCTTGTATGAGCTATTT R: CCTAATGAACGAACAAATTCTCCAAACTC
HKT1-Like	F: GATTCCTAACCCTGCAGATAAACCCATT R: GTTGCAGACACAGAGGTAAAGAACAAG
NHX1-Like	F: CACCAAAGAACTTGACAAGAATGCTG R: CCAATAGTAGTGCACGGTACGAG
ALMT9-Like	F: ACTTATGCAAAACTATACCCCACAATG R: TAGATAAACATATTCACCACCAAACACAC
PIP1-1-Like	F: CCCCAACAAGTGCTCCAGC R: CTTGGTGTAGCCGGAGCTG
PIP1-2-Like	F: TCACCTAGCAAGTGTGCCTCC R: CCTTGGTGTAGCCATGCTGC
PIP1-3-Like	F: GGCTCCCAACAAGTGTGCTT R: CCTTGGTGTAGCCATGTGCA
PIP1-4-Like	F: TCAGGTCTCCTACCAAGTGTGG R: CCCTTGGTATAGCCAGGGTTT
PIP2-1-Like	F: AAGTGAGCGAAGAGGGCCAAAC R: GTAGCGACGGTGACGTAGAGG
PIP2-2-Like	F: ATCTGAGCGAGGAAGGCCAAG R: GTGGCGACGGTGACGTAGAGA
PIP2-3-Like	F: CCACATTAACCCAGCAGTGACA R: CCGATGATCTCAGCTCCGAGT
PIP2-4-Like	F: AGGAAAGTGTCGCTGATCCGG R: TTGCTGTAGCCGTGAGCCACCGAA
PIP2-5-Like	F: GAGGAAGGTGTCGCTGATCAGA R: TGTTGTAGCCTGACGCCACCTCA
PIP2-6-Like	F: ATTGCTCTTCCTCTACGTCTCAGTG R: GCGACACCTTCCTGGCTAACAG

The first-strand cDNA was 30-fold diluted, using 1 μl as template in a final volume of 20 μl. Quantitative real-time PCR was performed on a StepOnePlus Real-Time PCR System (Life Technologies, Carlsbad, CA, USA), using SYBR premix Ex Taq (Tli RNaseH plus) (Takara Bio). The PCR protocol consisted of 10 min at 95°C, followed by 40 cycles of 15 s at 95°C, and 1 min at 60°C. The specificity of the reaction was assessed by the presence of a single peak in the dissociation curve and through size estimation of the amplified product by agarose electrophoresis. *DkACT* (Akagi et al., 2009) and *DkTUA* (Wang et al., 2016) were used as reference genes. The normalization factor was calculated by the geometric mean of the values of relative expression of both genes.

### Statistical Analyses and Raw Data

All the data analysis and graphics were made using RStudio v1.1.447 (2018) with packages from the Comprehensive R Archive Network (CRAN). Parameters for all the populations were statistically tested by Kruskal-Wallis test (P ≤ 0.05) and averages were compared with the Pairwise Wilcoxon-Mann-Whitney test at 95% confidence level (P ≤ 0.05) using the packages *dplyr* (Wickham et al., 2018), *ggplot2* (Wickham, 2016), *FSA* (Ogle et al., 2018), *DescTools* (Signorell, 2017), *rcompanion* (Mangiafico, 2018), *RColorBrewer* (Neuwirth, 2014), and *multcompView* (Graves et al., 2015). Principal component analysis (PCA) and correlogram were carried out using the packages *ggplot2* (Wickham, 2016), *factoextra* (Kassambara and Mundt, 2017), and *corrplot* (Wei and Simko, 2016). The variables included were the agro-morphological traits measured before the selection. A biplot of individual scores and loadings was obtained. For representation of the RNA expression data, packages *gplots* (Warnes et al., 2016) and *RColorBrewer* (Neuwirth, 2014) were used.

All the raw data has been deposited in https://github.com/fragimuo/Diospyros-salt-tolerance.

## Results

### Saline Stress Effect Among Populations

**Figure 1:**
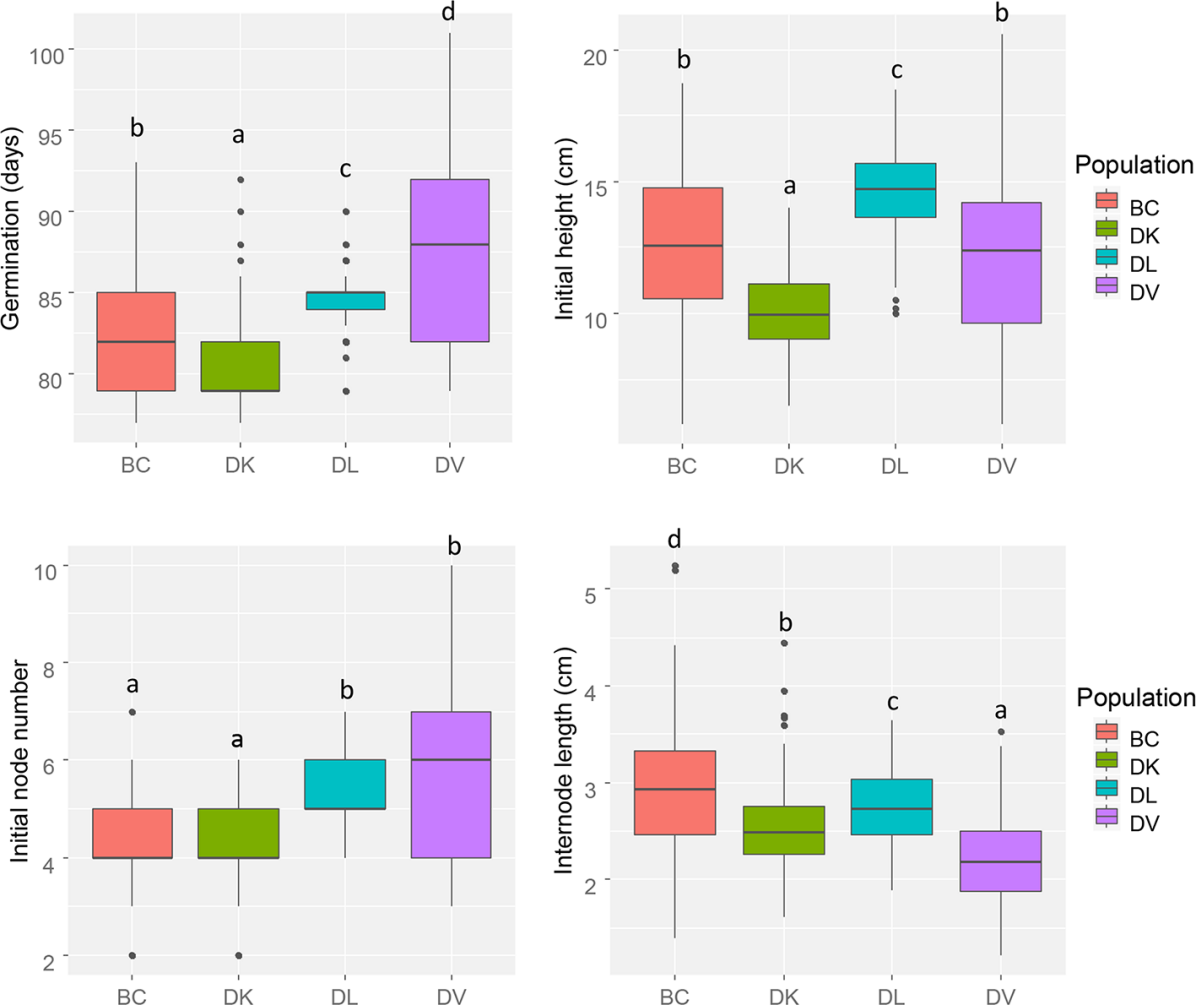
Box and whisker representation of initial morphologic parameters of the four populations (n = 128). Different letters mean statistical differences between populations.

Initial morphological parameters from the four populations were measured before salinity treatment began (**Figure 1**). The number of days from sowing to germination showed differences between all the populations. *D. kaki* (DK) population resulted in the smallest plants and *D. lotus* (DL) plants the tallest, backcross (BC), and *D. virginiana* (DV) populations had a similar initial height. No differences were observed in initial node number between DK and BC population and between DL and DV. Interestingly, the highest variability in initial phenotype was found in DV and BC populations compared to DK and DL populations.

**Figure 2:**
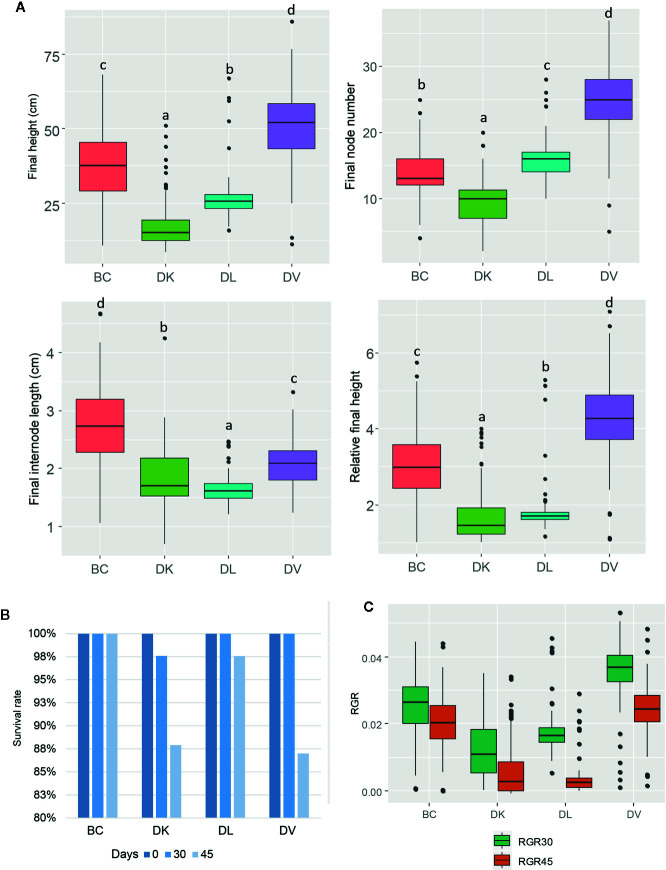
Box and whisker representation of morphologic parameters of the four populations (n = 128) after 45 days of salt treatment **(A)**. Different letters mean statistical differences between populations. Relative final height is the ratio between final and initial height. Percentage of plants that were still alive after 30 and 45 days of treatment **(B)**. Relative growth rate of 0–30 days and 30–45 days **(C)**.

**Figure 3:**
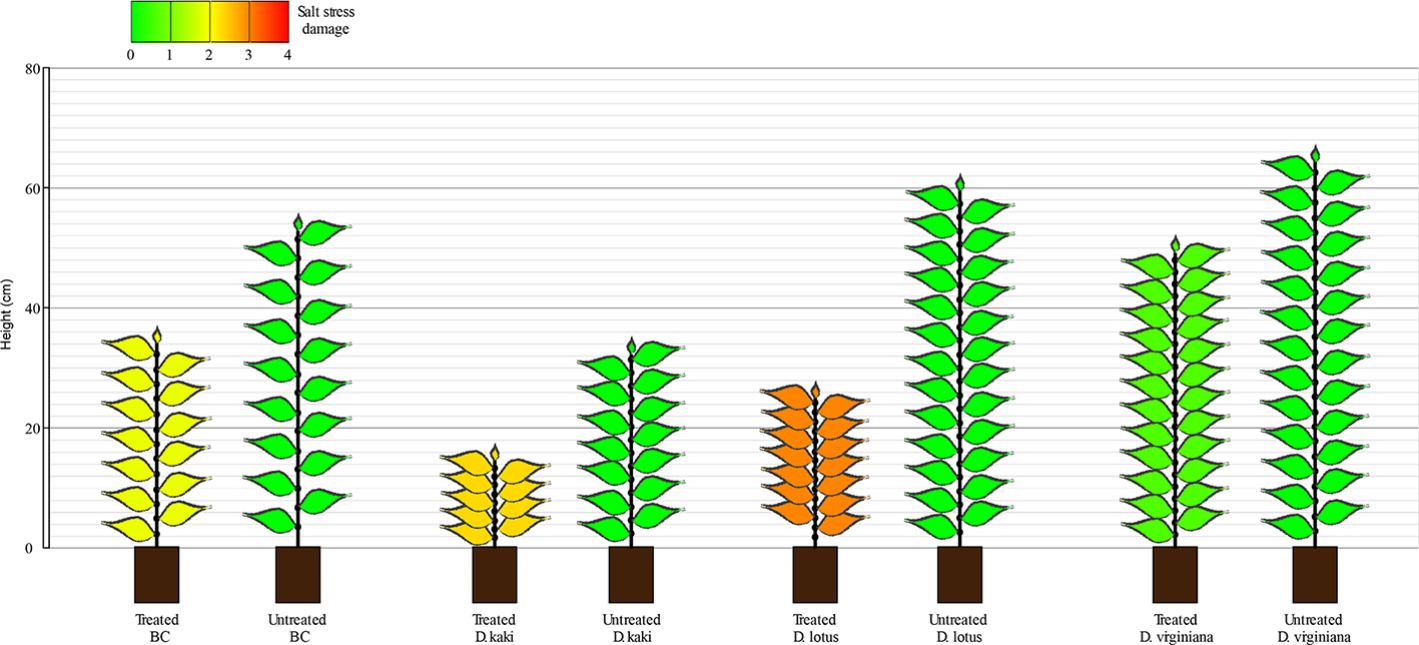
Representation of mean plants from each population with (n = 128) and without salt (control; n = 15 for DK, DV, and BC; n = 4 for DL) after 45 days of treatment (prior to plant selection). Height (cm) number of nodes, defoliation (as missing leaves), and salt stress damage are depicted.

After 45 days of saline stress, differences were noticeable among the four populations (**Figure 2**). DV and BC reached higher height than DL and DK populations. The BC population reached the highest internode length compared to other populations, suggesting higher vigor under salinity conditions. Again, more variability was observed among DV and BC populations compared to DK and DL regarding to morphological parameters. Furthermore, some of the plants did not survive to the salt stress treatment. The survival rate after 45 days of saline treatment was 100% for BC, 87% for DV, 89% for DK, and 98% for DL. Comparing the morphological traits before and after the saline treatment, DV and BC populations had less detrimental effects than DK and DL populations that showed significantly reduced height and severe salt stress damage (**Figure 3**).

**Figure 4:**
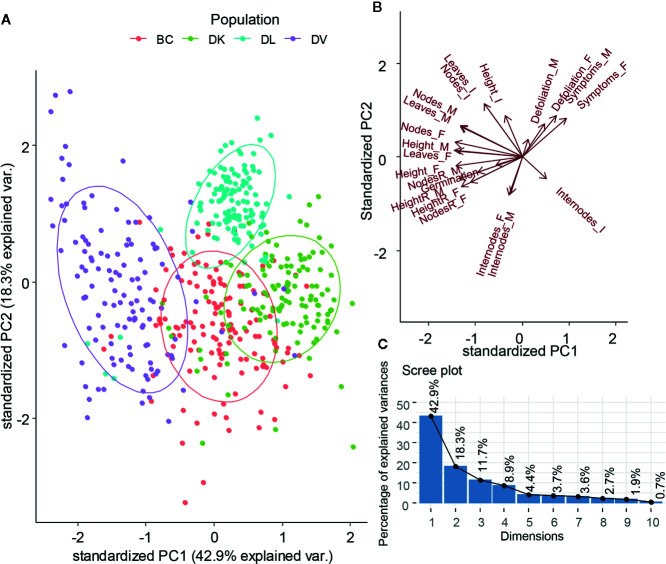
Plot of the first two components from Principal Component Analysis of the morphological variables **(A)**. Projection of the variables on the first two components **(B)**. Scree plot of the dimensions and explained variability for each component **(C)**.

All the morphological variables measured during the stress treatment were submitted to a Principal Component Analysis (PCA) (**Figure 4**). The first two components explained almost 60% of total variability. The PCA grouped clearly the individuals according to the population they belonged, but separated the populations DV, DK, and DL. The backcross population that combines genetic background from *D. kaki* and *D. virginiana* is located at equidistance from DK and DV populations. Furthermore, the DL dispersion was the narrowest. DV, BC, and DK population are separated by the PC1. The variables contributing to the PC1 were final plant height, final number of nodes, and relative final height (**Supplementary Figure 3**). DL population was separated from the other three by variables contributing to the PC2, final internode length, height growth, and defoliation.

### Saline Stress Tolerance Variability Within Populations

**Figure 5:**
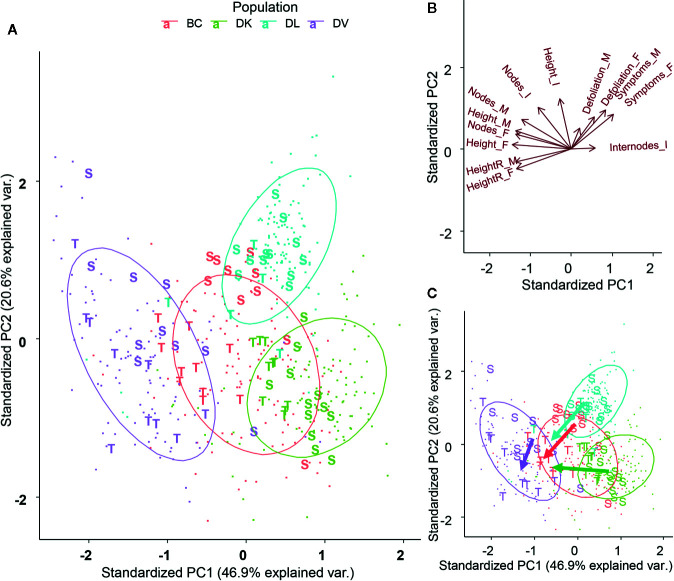
Plot of the first two components from Principal Component (PC) Analysis of the morphological variables after removal of highly collinear variables **(A)**. Plants selected as Tolerant and Sensitive according its phenotype have been represented. Projection of the variables on the first two components **(B)**. Dimensional division between tolerant and sensitive subsets for each population **(C)**.

Using the symptoms and overall plant status, subsets of tolerant and sensitive plants were chosen from each population. The observed symptoms and the scale used are shown on **Supplementary Figure 2**. The tolerant plants were a subset of plants showing less damage caused by salt, and the sensitive ones were the most affected within the population’s species. These subsets were submitted to a PCA using the morphological variables, but removing those that showed a high collinearity in the previous PCA (**Figure 5A**). In the four populations sensitive and tolerant plants were grouped apart. DK, BC, and DL were separated on similar directions, according to the PCA variables final height and height growth (**Figure 5B**). DV population showed less clear separation between tolerant and sensitive plants, mainly in the PC2 axis. All the populations showed a coherency in the separation between tolerant and sensitive plants (**Figure 5C**), which implies that the morphological traits used are a useful tool for phenotyping salt tolerance. Due to the higher tolerance to salinity in the DV population, the discrimination by phenotype was weaker.

**Figure 6:**
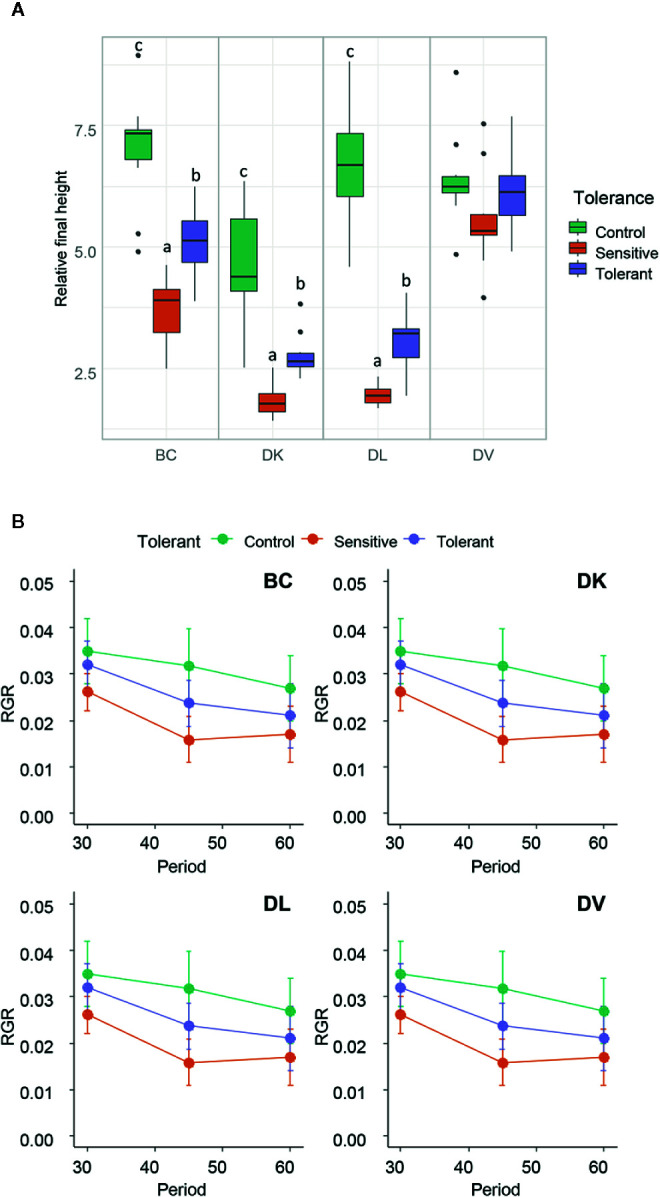
**(A)** Box and whisker representation of relative final height (ratio between final and initial) of the four populations after 60 days of salt treatment. Individuals from each population were grouped into tolerant and sensitive according to its phenotype as detailed in *Methods*. Control plants were included. Different letters mean statistical differences between populations. **(B)** Representation of the Relative Growth Ratio (RGR) for the subsets of the four populations in the periods of 0–30, 30–45, and 45–60 days. Values are calculated using several biological replicates for control (10, 10, 4, and 10), tolerant (10, 10, 5, and 10), and sensitive (10, 12, 12, and 10) BC, DK, DL, DV, respectively.

A correlogram using the morphological variables was done, including the leaf damage symptoms (**Supplementary Figure 4**). The height growth parameter and the relative final height were the variables more correlated with final salt damage symptoms. Differences for both variables were found between tolerant and sensitive individuals in the DK, DL, and BC populations (**Figure 6**). Furthermore, in these populations, the control plants had higher values than both the tolerant and sensitive subsets, except for the DK population in which the control plants were statistical identical to the tolerant subset regarding to height. No significant differences were found within the DV population.

### Physiological and Nutritional Effects Related to Salinity Tolerance

In order to elucidate the salinity tolerance mechanisms within each species and population, once the populations were divided into subsets of tolerant and sensitive plants to saline stress, we analyzed plant water status, leaf gas exchange, and nutritional parameters for each subset after 60 days of saline stress.

**Figure 7:**
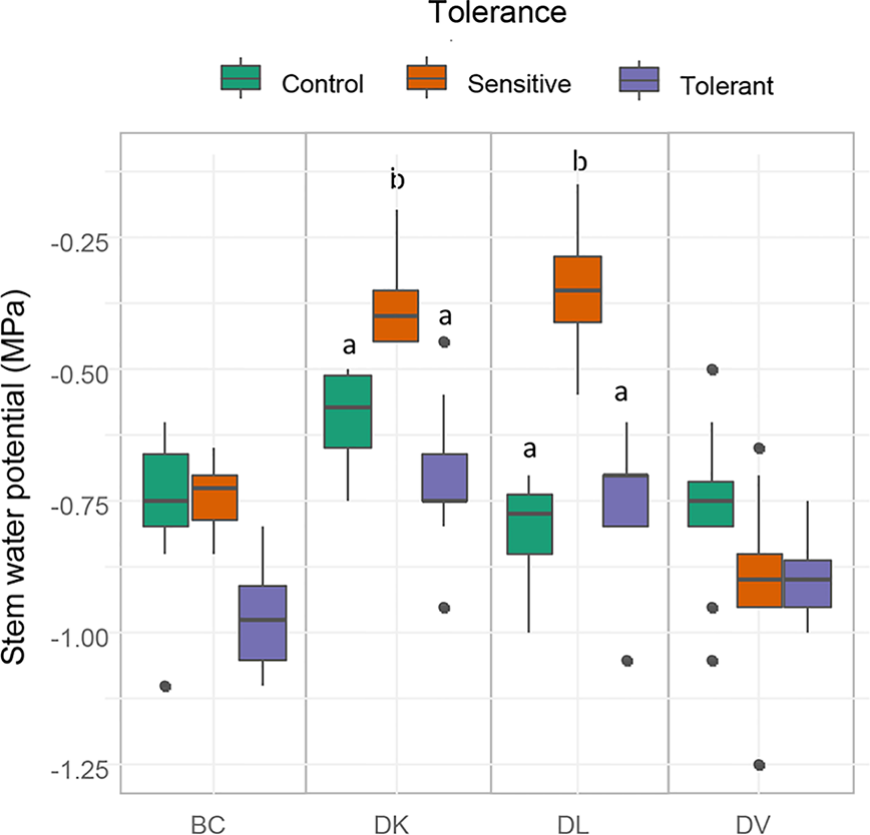
Box and whisker representation of stem water potential of the four populations after 60 days of salt treatment. Genotypes were grouped into tolerant and sensitive according to its phenotype. Control plants were included. Different letters mean statistical differences between populations. Values are calculated using several biological replicates for control (10, 10, 4, and 10), tolerant (10, 10, 5, and 10), and sensitive (10, 12, 12, and 10) BC, DK, DL, DV, respectively.

**Figure 8:**
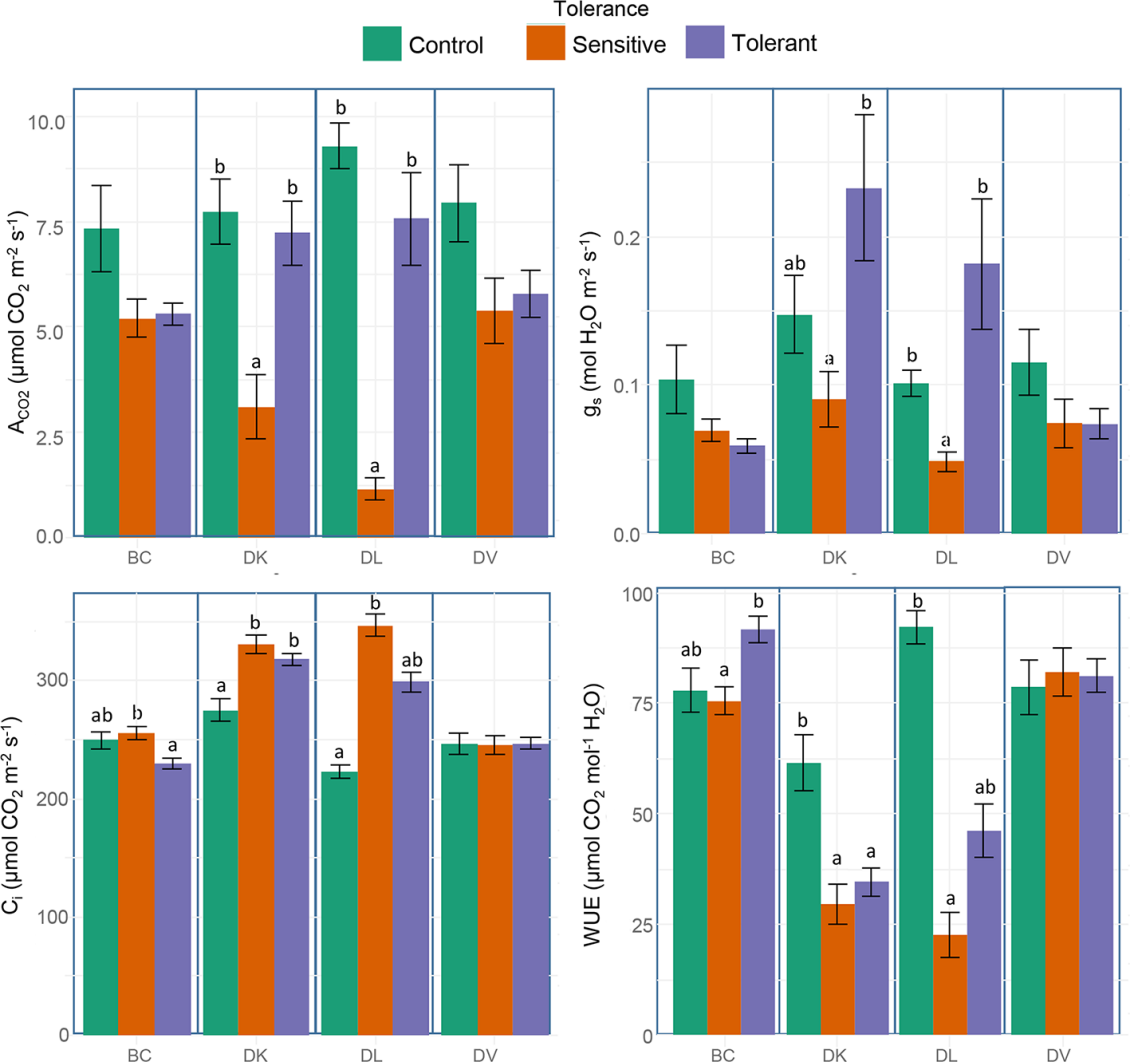
Box and whisker representation of leaf net CO_2_ assimilation rate (A_CO2_), stomatal conductance (g_s_), internal CO_2_ concentration (C_i_), and intrinsic water use efficiency (WUE) of the four populations after 60 days of salt treatment. Genotypes were grouped into tolerant and sensitive according to its phenotype. Control plants were included. Different letters mean statistical differences between populations. Values are calculated using several biological replicates for control (10, 10, 4, and 10), tolerant (10, 9, 5, and 10), and sensitive (10, 12, 11, and 10) BC, DK, DL, DV, respectively.

Stem water potential was measured to determine differences in plant water status between the different populations. Sensitive plants showed significant better plant water status (higher values of stem water potential) than the tolerant in both DK and DL populations (**Figure 7**). In addition, in these populations, the values of stem water potential from the tolerant subset and the control were statistically identical. Respect to the leaf gas exchange parameters, all tolerant populations maintained similar values of A_CO2_ and g_s_ than control plants, whereas in sensitive subsets from DK and DL were significantly decreased (**Figures 8A, B**). The C_i_ were significantly higher in both sensitive and tolerant DK and DL populations than in control plants (**Figure 8C**). In the case of BC populations, C_i_ were significantly lower in tolerant than in sensitive plants, whereas C_i_ remained unaltered in DV plants. On the other hand, the DK and DL populations showed significantly lower WUE in both tolerant and sensitive plants than in the control (**Figure 8D**). The BC population had higher WUE in tolerant plants than in sensitive. No differences were observed between any population subset of DV.

**Figure 9:**
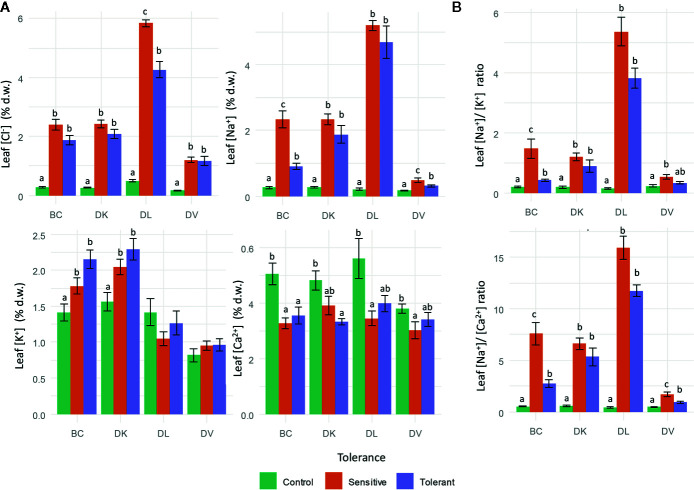
Leaf ion content measurements after 60 days of salinity treatment. Cl^−^, Na^+^, K^+^, and Ca^2+^ **(A)** and Na^+^/K^+^ and Na^+^/Ca^2+^ ratios **(B)**. Genotypes were grouped into tolerant and sensitive according to its phenotype. Control plants were included. Different letters mean statistical differences between populations. Values are calculated using several biological replicates for control (10, 10, 4, and 10), tolerant (9, 9, 5, and 10), and sensitive (10, 10, 12, and 9) BC, DK, DL, DV, respectively.

Leaf ions content were analyzed to quantify toxic ion accumulation (Cl^−^ and Na^+^) and nutritional imbalances (K^+^ and Ca^2+^) caused by saline stress. Leaf Cl^−^ accumulation significantly increased in both sensitive and tolerant populations (**Figure 9A**), but in DL populations sensitive plants had higher leaf Cl^−^ content than tolerant plants (**Table 2**). Regarding to leaf Na^+^ accumulation, on DV and BC populations, Na^+^ foliar content was higher on sensitive than tolerant subsets (**Table 2**; **Figure 9B**), whereas on DK and DL populations no differences were observed between tolerant and sensitive subsets. Comparing leaf Na^+^ accumulation between populations, DV showed the lowest values. Control plants had lower Na^+^ content in all populations (**Figure 9B**).

**Table 2:** Relative ionic content of each tolerant and sensitive populations compared to control.

	BC		*D. kaki*		*D. lotus*		*D. virginiana*	
	Tolerant	Sensitive	Tolerant	Sensitive	Tolerant	Sensitive	Tolerant	Sensitive
**Cl^−^**	+	+	+	+	+	++	+	+
**Na^+^**	+	++	+	+	+	+	+	++
**K^+^**	++	+	+	+	=	=	=	=
**Ca^2+^**	–	–	–	=	=	–	=	–

Two signs refer to statistically different concentrations between tolerant and sensitive.

Analyzing the nutritional imbalances caused by salinity, the K^+^ content in the BC population was higher in tolerant plants than sensitive ones, being both significantly higher than the control plants. However, in the DK a lower K^+^ is observed in the control plants compared to the sensitive and tolerant subsets. No significant differences are observed in DL and DV populations (**Table 2**). Regarding to Ca^2+^ content, it was lower in tolerant plants than in control, but without differences with the sensitive subset. Nutrient balances were also calculated for Na^+^/K^+^, Na^+^/Ca^2+^. The Na^+^/K^+^ balance increased in all salinized plants, but differences between tolerant and sensitive plants were only observed in the BC population (**Figure 9B**). In all populations the control plants had the lowest Na^+^/K^+^ ratio. Similar results were obtained for Na^+^/Ca^2+^, except that in DV population differences were observed among all subsets.

### Differential Expression of Genes Related to Salinity Tolerance

**Figure 10:**
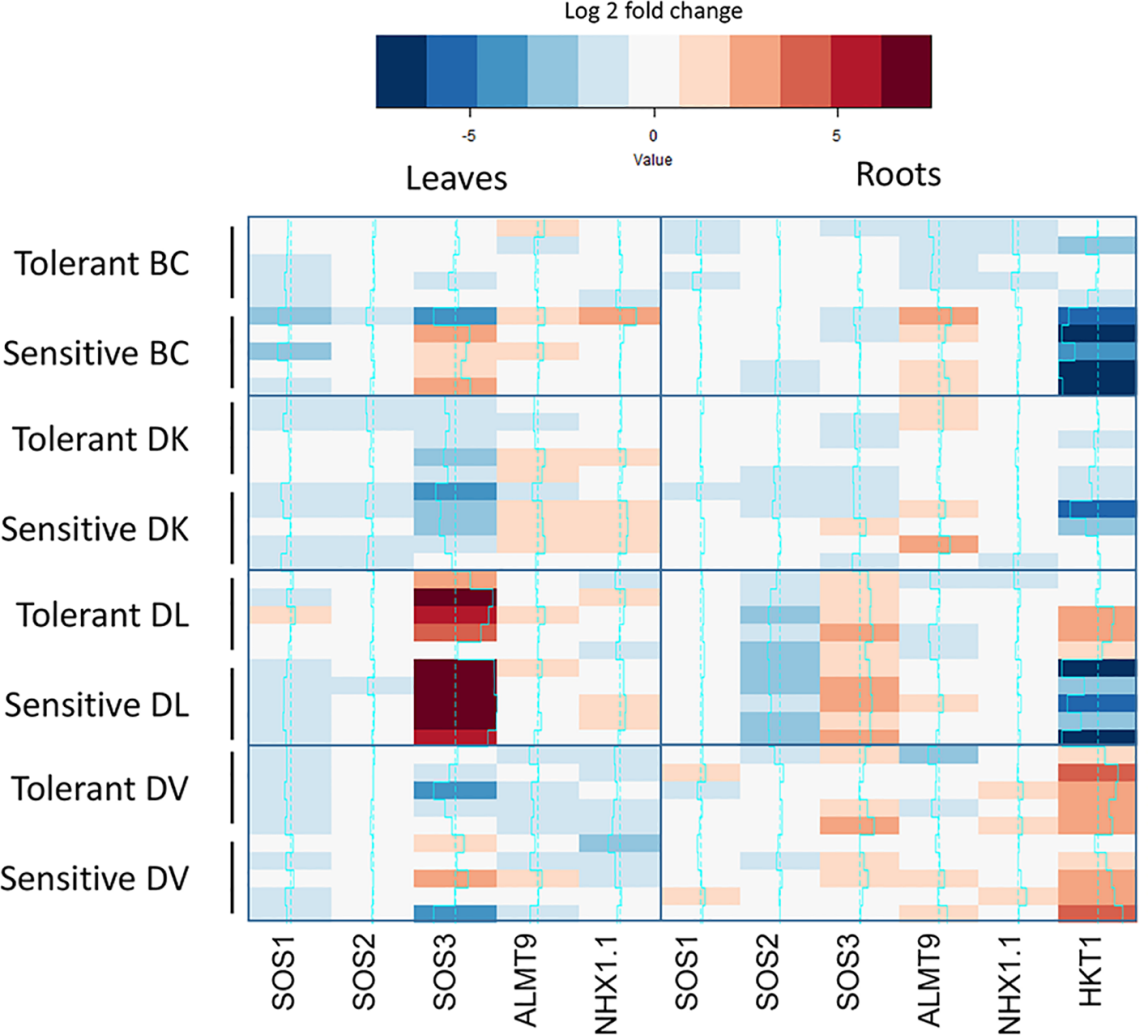
Expression analysis of salt tolerance related-like genes from control and treated subsets of tolerant and sensitive plants from each population. Expression is mean value of three biological replicates of each individual. Only significant differences between control and treated subsets are show in color.

Differential expression of genes related to salt-tolerance was analyzed in leaves and roots of five individuals of each subset at the end of the salt treatment (60 days). Control plants were included in the analysis (**Supplementary Figure 5**). Leaf expression of *SOS1-like*, *SOS2-like* and ALMT9-like was similar among all the populations and different in root tissue. *HKT1-like* expression on leaf was undetectable in all populations, but detectable in roots, being the lowest expression in DL. On the other hand, *NHX1.1-like* highest expression in leaf corresponded to DV population and in roots to BC. In order to compare between tolerant and sensitive subsets, all gene expression was relative to the control plants of each population and a heatmap was constructed using the Log2fold change (**Figure 10**). On leaves, SOS3-like expression was downregulated on both subsets of DK population under salt treatment, whereas an upregulation was observed in the DL population. Interestingly, an upregulation was observed only in the sensitive subset of the BC population on 4/5 plants. Regarding to roots, *SOS2-like* expression was downregulated under salt treatment on the BC population, whereas *SOS3-like* was upregulated. *ALMT9-like* gene expression was upregulated on sensitive BC subset. About *HKT1-like* gene expression, a downregulation has observed in sensitive subset of BC and DL populations, whereas in tolerant DL subset and DV population a higher expression has been observed.

**Figure 11:**
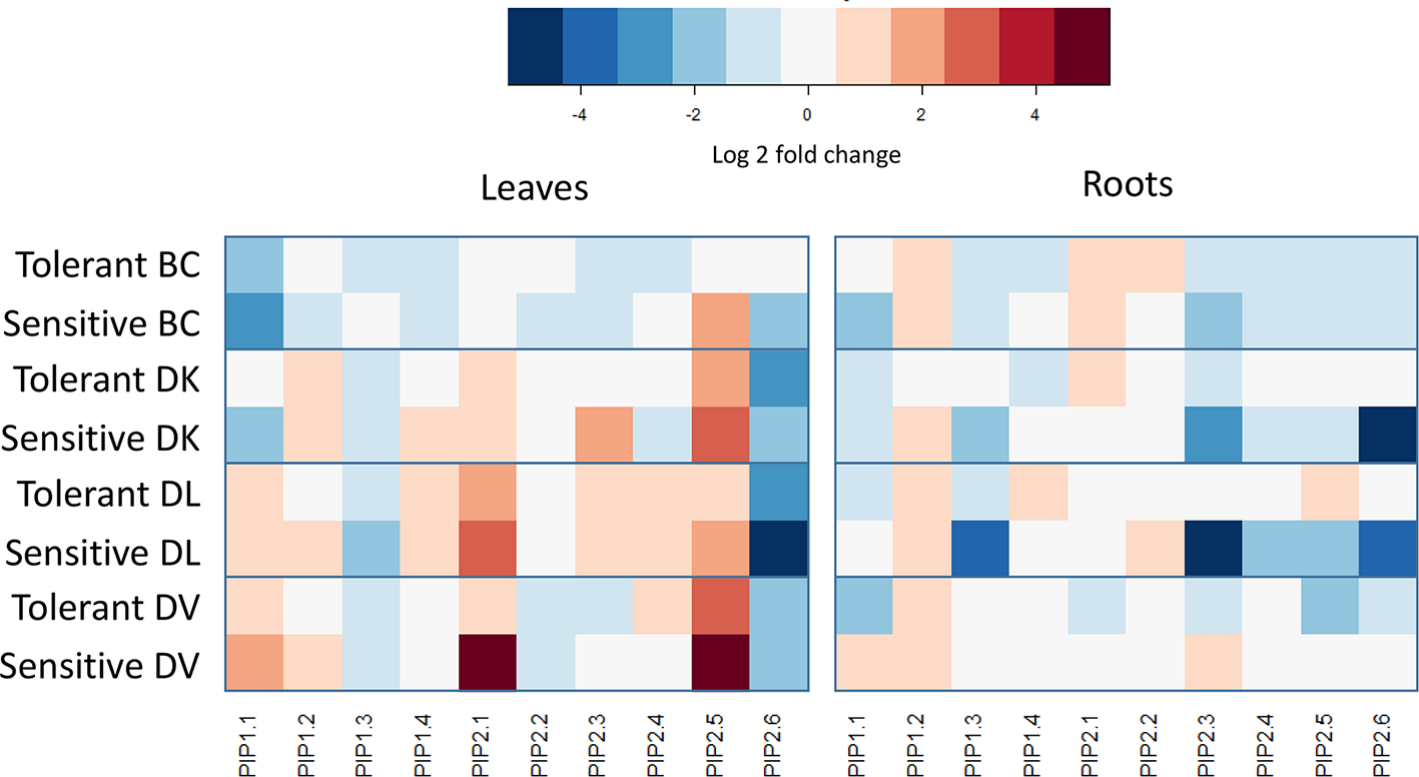
PIP aquaporin-like genes expression changes between control and treated subsets of tolerant and sensitive plants from each population. Expression values were calculated using three biological replicates from each group. Only significant differences between control and treated subsets are colored.

After obtaining the sequences of the putative orthologues of *PIPs* in *Diospyros lotus*, a phylogenetic tree was constructed (**Supplementary Figure 6**). *PIP1-like* family of *D. lotus* was grouped together with the *PIP1* *Arabidopsis thaliana* genes, whereas *D. lotus* *PIP2-like* genes were grouped with the *A. thaliana* *PIP2* genes. Expression of *PIP1-like* and *PIP2-like* aquaporin families were analyzed and compared between tolerant and sensitive subset bulks for each population. A heat map was constructed using the control bulk for normalizing within populations (**Figure 11**). On the BC population, *PIP2-like 5* showed a four-fold upregulation on leaves from sensitive plants compared to tolerant, Furthermore, *PIP2-like 6* and *PIP1-like 1* showed a downregulation in sensitive plants compared to tolerant in leaves and roots, respectively. For these genes, the tolerant plants had similar levels of expression to the control plants. Regarding to the DK population; comparing sensitive to tolerant leaves, *PIP1-like 1* and *PIP2-like 5* showed a downregulation, whereas *PIP2-like 3* showed an upregulation. In roots, a strong (32 fold) downregulation was shown in *PIP2-like 6*. Both *PIP1-like 3* and *PIP2-like 3* presented a downregulation in sensitive plants. The DL population showed similar expression differences in roots between tolerant and sensitive to the DK population. In addition, *PIP2-like 5* showed less expression in sensitive roots. In leaves of DV population, the only differential *PIP-like* expression between tolerant and sensitive plants was in *PIP2-like 1*, where the sensitive plants showed a strong upregulation. On roots, *PIP1-like 1*, *PIP2-like 3* and *PIP2-like 5* exhibited lower expression in tolerant plants. Regarding to the salt induction of this genes, *PIP1-like 1* has shown opposite expression patterns in the BC and DK population than the DL and DV populations.

## Discussion

### Saline Stress Effect Among Populations

The severity of the symptoms in the different populations is coherent with the described differences between these species regarding to salinity tolerance, being *D. virginiana* the most tolerant specie among populations followed by *D. kaki* and *D. lotus* (de Paz et al., 2016b; Visconti et al., 2017; Gil-Muñoz et al., 2018a). The salinity stress results show that the *D. kaki* most compatible rootstock species (*D. kaki*, *D.* *lotus* and *D. virginiana*) have much lower salt stress tolerance than other *Diospyros* species such as black sapote (*D. digyna*), that even does not show leaf burn when exposed to 16 dS m^-1^ for more than 17 weeks (Mickelbart and Marler, 1998). All the populations exhibited a reduction in the internode length compared to the control. This effect is believed to be caused by the salinity stress response mechanism mediated by DELLA proteins (Achard et al., 2006) as adaption to both saline and osmotic stress *via* ROS detoxification (Achard et al., 2008). The side effect of DELLA expression would be a reduction in bioactive GAs (Achard et al., 2006; Magome et al., 2008) causing a reduction in the internode length.

### Saline Stress Tolerance Variability Within Populations

Differences related to the variability of morphological parameters among the three populations studied were obtained. Furthermore, the Principal Component Analysis (PCA), showed the widest distribution in the DV population, whereas the narrowest corresponded to the DL population. Differences in variability can be explained by the genetic structure of each population; DV population comes from an open pollination family, whereas DL is formed by full siblings. Interestingly, DK population is an open pollination family, but shows narrower variability than DV population. This fact can be explained by the low genetic variability previously described for *D. kaki* (Naval et al., 2010; Parfitt et al., 2015; Gil-Muñoz et al., 2018b). BC population shows an intermediate variability between DV and DK as expected because it combines both genetic backgrounds.

The variability observed in the analyzed parameters is consistent with the population genetic structures. This fact can indicate that a significant percentage of the observed variability might be controlled by genetic traits. The PCA analysis has been also consistent with the genetic structure of the populations, and it can be a useful tool to aid selection during a plant breeding program, as it extracts, compresses, simplifies, and analyzes complex multidimensional datasets (Das et al., 2017). Furthermore, with large datasets, some collinearity is expected to be present among the measured variables. Thus, measurement errors and phenotyping noise can be reduced by PCA projection, providing more precise individual phenotyping. Furthermore, the PCA can provide the importance of easy-measurable variables to the overall desired trait, giving a powerful aid in the selection.

### Physiological and Nutritional Effects Related to Salinity Tolerance

Differences in vegetative development between tolerant and sensitive subsets in both DL and DK populations can be explained by the effect of a poor adaption to the osmotic stress, as shown by changes in plant water status and the lower photosynthesis rate. However, which one is the cause and which one the effect is not yet known (Munns and Tester, 2008). Nevertheless, ABA contributes to stress tolerance by regulating water balance and stomatal closure (Lim et al., 2015). However, our results show higher stomatal closure in sensitive than in tolerant plants, indicating that the sensitive populations have suffered higher saline stress. On the other hand, differences in photosynthesis, stomatal aperture, or water relations between tolerant and control plants are not observed, indicating that the tolerant subsets had a better response to the osmotic stress than the sensitive subsets in both populations. Furthermore, differences were neither observed between tolerant and control final internode relative length, discarding DELLA response as a mechanism effect. All facts indicate that the observed differences in growth rate and relative final height between tolerant and control plants must be mainly related to ionic stress, which affects growth in a different way than osmotic stress (Munns and Tester, 2008). Respect to the BC population, no differences in parameters as relative growth and growth rate, photosynthesis, stomatal aperture, or water relations were obtained in the analyzed subsets. This may indicate that both tolerant and sensitive plants are not strongly affected by the osmotic stress but rather the ionic stress and differences between these groups might be related to ion exclusion. The DV population has proven to be highly tolerant to saline stress as previously described (de Paz et al., 2016b; Gil-Muñoz et al., 2018a). The similar growth rates and relative final weight among the subsets while maintaining photosynthesis, stomatal aperture, and plant water status indicate the presence of highly efficient mechanisms to overcome osmotic and saline stress.

As foliar contents of Na^+^ and Cl^-^ have been analyzed, other mechanisms such as vacuolar sequestration have not been detected that could further explain the differences observed. Our aim has been to study intake and root to shoot exclusion mechanisms taking into account the background objective of breeding rootstock plant, as different scions might have different responses mechanisms of vacuolar ion sequestration. Variability in osmotic stress tolerance is present within DK, DL, and BC populations and in Cl^−^ uptake within DL population. The differences for Na^+^ exclusion observed within BC population but not in DV and DK populations indicate that DV and DK populations have different Na^+^ exclusion mechanisms, being the DV mechanism more efficient. Further crosses between DL and DV might reveal variability in Cl^−^ exclusion mechanisms that might help to untangle the salt tolerance mechanisms present in *Diospyros* genus.

### Differential Expression of Genes Related to Salinity Tolerance

Regarding to salt tolerance-related genes expression, a strong upregulation of *SOS3-like* gene has been observed in the DL population on both tolerant and sensitive plants. This gene is responsible for activating the SOS salinity response mechanism when a low Ca^2+^ concentration is detected in the cell (Gong et al., 2004). Interestingly, DL population showed similar Na^+^ accumulation on leaves from control plants than leaves from the end of the salinity experiment. This population presented the highest Na^+^ concentration in leaves and both the highest Na^+^/K^+^ and Na^+^/Ca^2+^. Furthermore, the higher *SOS3-like* expression present in the sensitive plants leaves of the BC population, but not in the DK in despite of similar Na^+^, Ca^2+^ and Na^+^/Ca^2+^ levels are observed in the DK population and BC sensitive plants. Surprisingly, differences in *SOS3-like* response are present even though 75% of the BC population genome comes from DK population. *SOS3-like* expression level is upregulated on plants where it can be a response caused by the toxic Na^+^ concentrations in leaves and not a direct indicator of salinity tolerance, s. BC population might have inherited the more sensitive SOS response mechanism present on DV population, whose response has not been observed due to the low levels of Na^+^ in leaves. On the other hand, *HKT1-like* gene expression differences on roots observed between tolerant and sensitive subsets in BC population were consequent with the tolerant/sensitive phenotype similarly to the observations made in other species (Arabbeigi et al., 2018). *HKT1* higher expression could contribute to the salinity tolerance, as this gene is responsible to avoid Na^+^ translocation from roots to shoot *via* xylem transport (Hanin et al., 2016). PIP gene expression has been linked to an increase in osmotic stress tolerance (Katsuhara et al., 2014; Afzal et al., 2016; Alavilli et al., 2016). In our study, a downregulation in several PIPs has been observed in roots of sensitive subsets of both DL and DK populations where lower stem water potential values were measured. Furthermore, a strong upregulation was observed in some *PIP2-like* family members on leaves of the sensitive DV subset. In Arabidopsis, the AtPIP2.1 gene has been described to act as a Na^+^ gate to the leaves (Chaumont and Tyerman, 2014).

To sum up, in DK population, we have not observed variability in active Na^+^ or Cl^−^ exclusion mechanisms, but they are present for osmotic stress tolerance *via* water balance regulation. Regarding to the DL population, we have observed the lowest basal expressions of *SOS1-like* gene and *HKT1-like* in roots compared to the other populations. Also, this population has poor ability to exclude Na^+^ ions and therefore prevent toxic concentrations. However, variability in both osmotic stress tolerance *via* water balance regulation is present. Cl^−^ exclusion variability in this population has been observed, but the mechanism is yet unknown. The DV population tolerance to salinity has been also linked to higher *SOS1-like* basal expression and *HKT1-like* upregulation in roots. In other species, the role of this gene is linked to avoid Na^+^ translocation from roots to shoot *via* xylem transport (Hanin et al., 2016) to the prevention of. Furthermore, in a previous study we observed a higher root expression of HKT1-like gene in a salt-tolerant subset of a *Diospyros* population (Gil-Muñoz et al., 2020). In addition, variability in the indicators used in this work to identify mechanisms related to salt stress tolerance are not observed within this population.

### Conclusions

Among the different populations studied, different mechanism of tolerance could be hypothesized, providing different strategies for breeding for salinity tolerance.

### BC Tolerant Population

The mechanism of tolerance to osmotic stress found was based in the water use efficiency (WUE). BC tolerant population has shown to have better tolerance to saline stress than sensitive BC through a higher WUE, which agrees with previous results on *Diospyrus* genus (Gil-Muñoz et al., 2020). Since the lack of variability for this character in the DV and DK populations, we can conclude the variability into BC has been generated through crossing. We have generated a tolerant population by back crossing in which the water use efficiency is higher than in DV population, which might be a sign of transgressive segregation through expressing the DV higher tolerance under the DK genetic background. Concerning to ionic stress, the Na^+^ accumulation was higher in the BC than in the DV population, indicating other factors involved in Na^+^ absorption. The expression of the genes analyzed resulted in a high expression of *HKT1-like* in roots of DV population; this gene is being involved in Na^+^ exclusion, which might be a mechanism of preventing Na^+^ translocation to the higher parts of the plant in *D. virginiana* species.

### DK Population

Little variability was found within the DK population regarding to salinity tolerance, neither by ion leaf content nor salt tolerance gene expression. In this case, the observed differences between tolerant and sensitive subsets might be explained by differences in osmotic stress tolerance, but not in ionic stress. In this population, the tolerance might be based only in plant water regulation *via* stomatal aperture and water flow.

### DL Population

Similarly, to the DK population, the tolerance observed in DL population for Na^+^ might be based in passive mechanisms through water regulation. In despite of DL population was the most susceptible to salinity, is the only population that has shown differences in Cl^−^ accumulation. However, with the analyzed data we cannot provide a hypothesis that can explain this fact, as water flow differences were similar than the observed in DK population and this differences in Cl^−^ accumulation were not detected. Also, the expression analysis revealed high expression of *HKT1-like* in roots of the tolerant subset despite of it cannot be linked to lower Na^+^ leaf content.

### DV Population

In this population, variability for saline tolerance was low, most of the plants showed high saline tolerance. Significant Na^+^ accumulation might be related to the higher *PIP2-like* family expression in leaves, acting as a gate of Na^+^ influx into the leaves.

As a result of the comparison between physiological parameters, transcriptomic analysis and phenotypes under saline conditions, we can conclude that interspecific crosses of *D. virginiana* can be an option for breeding persimmon rootstocks tolerant to salinity. Crosses with *D. kaki* can provide better rootstock-scion compatibility with varieties than genotypes from *D. virginiana*. However, further selection of agronomic traits that improve crop management would be most necessary.

## Data Availability Statement

All datasets generated for this study are included in the article/**Supplementary Material**.

## Author Contributions

MB and MN contributed conception and design of study. FG-M performed the experiments, and data analysis. AQ contributed to the data. JP-P and MB contributed to data analysis and interpretation. MB provided supplies and facilities. FG-M wrote the first draft of the manuscript. MB and JP-P revised the manuscript. All authors contributed to the article and approved the submitted version.

## Funding

JP-P gratefully acknowledges the postdoctoral contract “Ramón y Cajal” program (RYC-2015-17726), supplied by the Spanish Ministry of Economy, Industry and Competitiveness (MINECO).

FG-M acknowledges the support granted with the PhD fellowship co-financed by the European Social Fund and the Generalitat Valenciana ACIF/2016/115). The project was partially funded by the IVIA grant 51914 and FEDER funds.

## Conflict of Interest

The authors declare that the research was conducted in the absence of any commercial or financial relationships that could be construed as a potential conflict of interest.
