# Supplementary material for: Intra and Inter-specific Variability of Salt Tolerance Mechanisms in Diospyros Genus
Source: Front Plant Sci. 2020 Aug 7;11:1132. doi: 10.3389/fpls.2020.01132 (PMC7427203; doi:10.3389/fpls.2020.01132)
Supplement: Supplementary file 1 [file Presentation_1.pptx]

## Slide 1
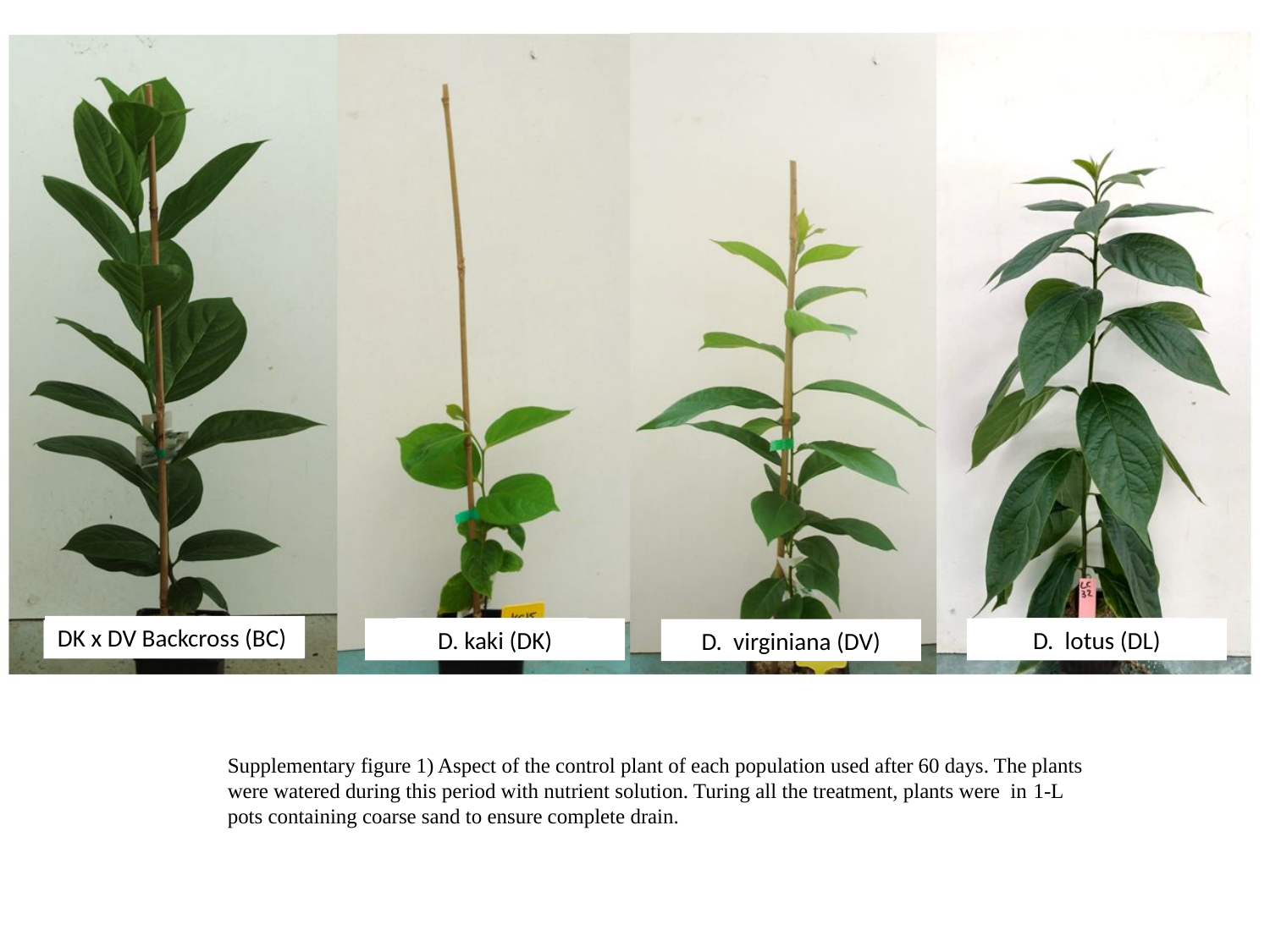

DK x DV Backcross (BC)
D. kaki (DK)
D. lotus (DL)
D. virginiana (DV)
Supplementary figure 1) Aspect of the control plant of each population used after 60 days. The plants were watered during this period with nutrient solution. Turing all the treatment, plants were in 1-L pots containing coarse sand to ensure complete drain.

## Slide 2
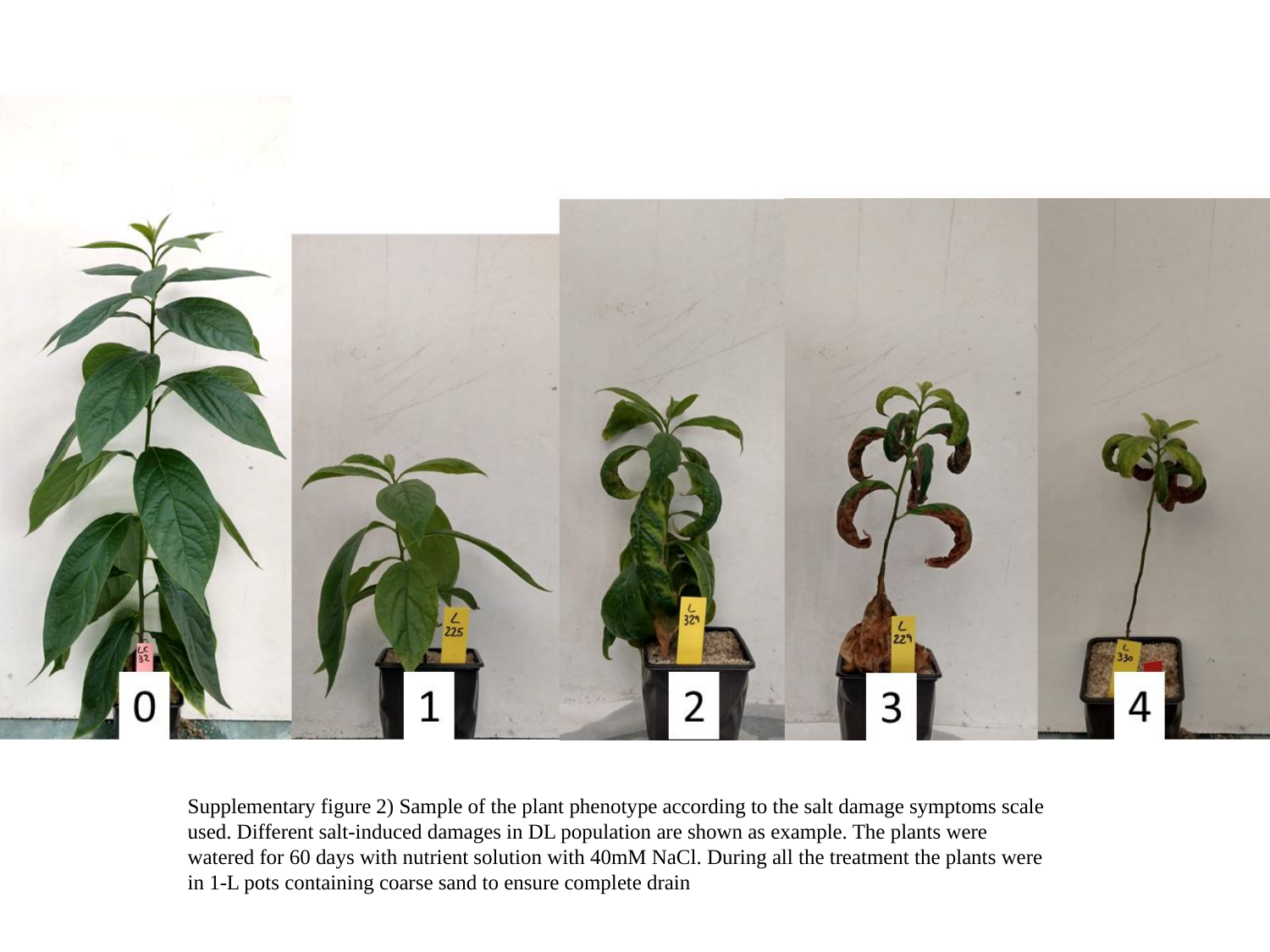

Supplementary figure 2) Sample of the plant phenotype according to the salt damage symptoms scale used. Different salt-induced damages in DL population are shown as example. The plants were watered for 60 days with nutrient solution with 40mM NaCl. During all the treatment the plants were in 1-L pots containing coarse sand to ensure complete drain

## Slide 3
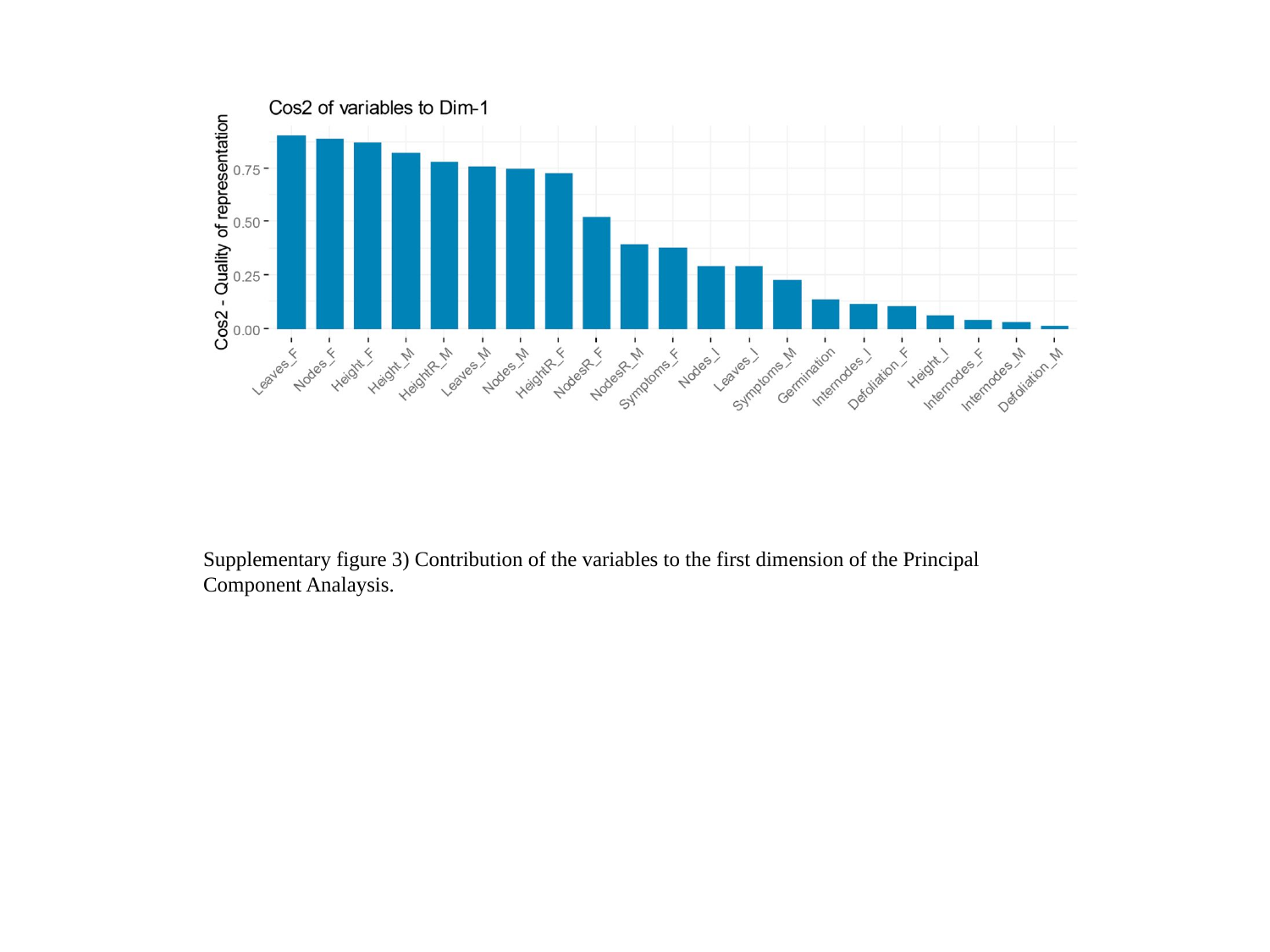

Supplementary figure 3) Contribution of the variables to the first dimension of the Principal Component Analaysis.

## Slide 4
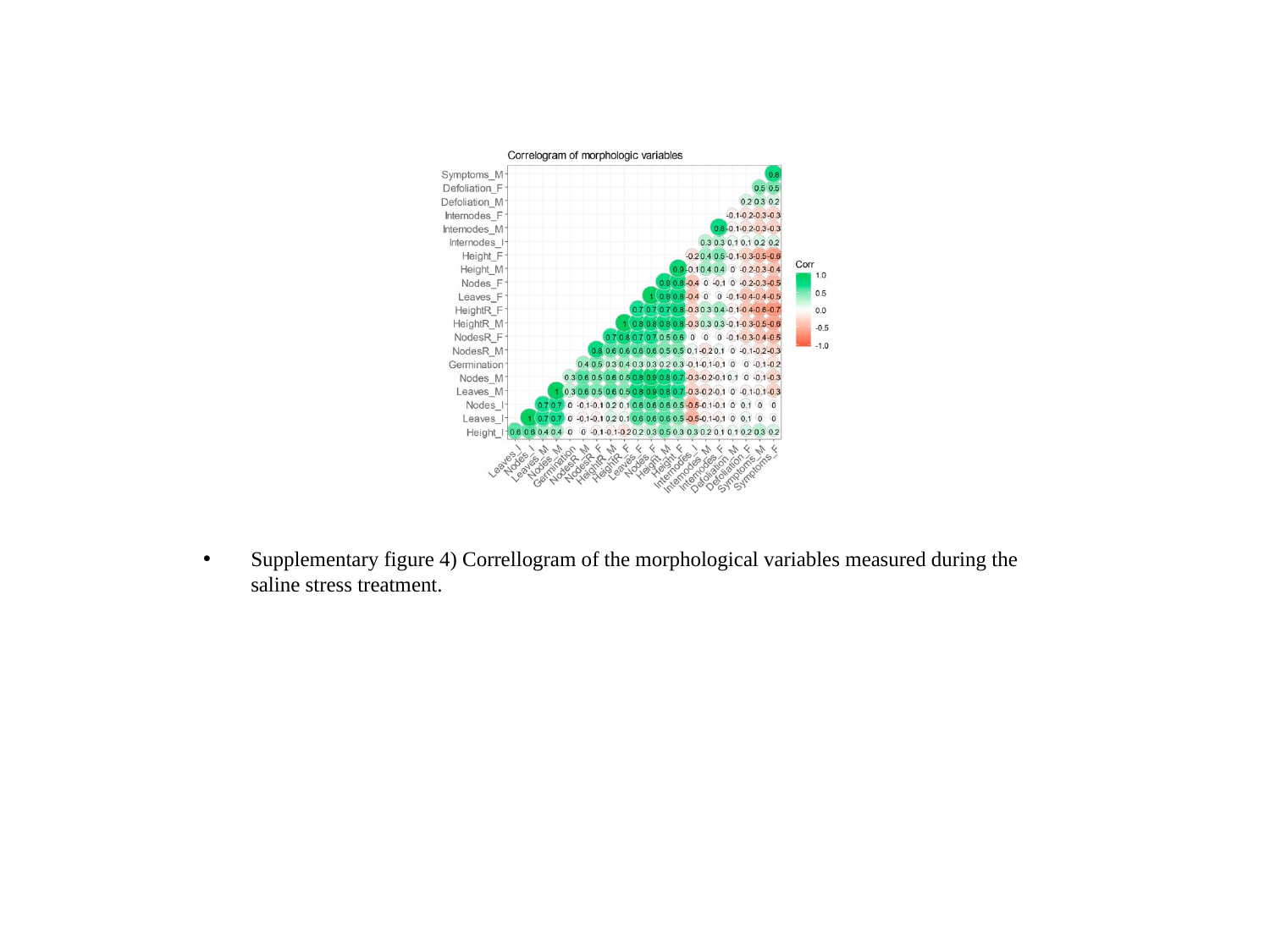

Supplementary figure 4) Correllogram of the morphological variables measured during the saline stress treatment.

## Slide 5
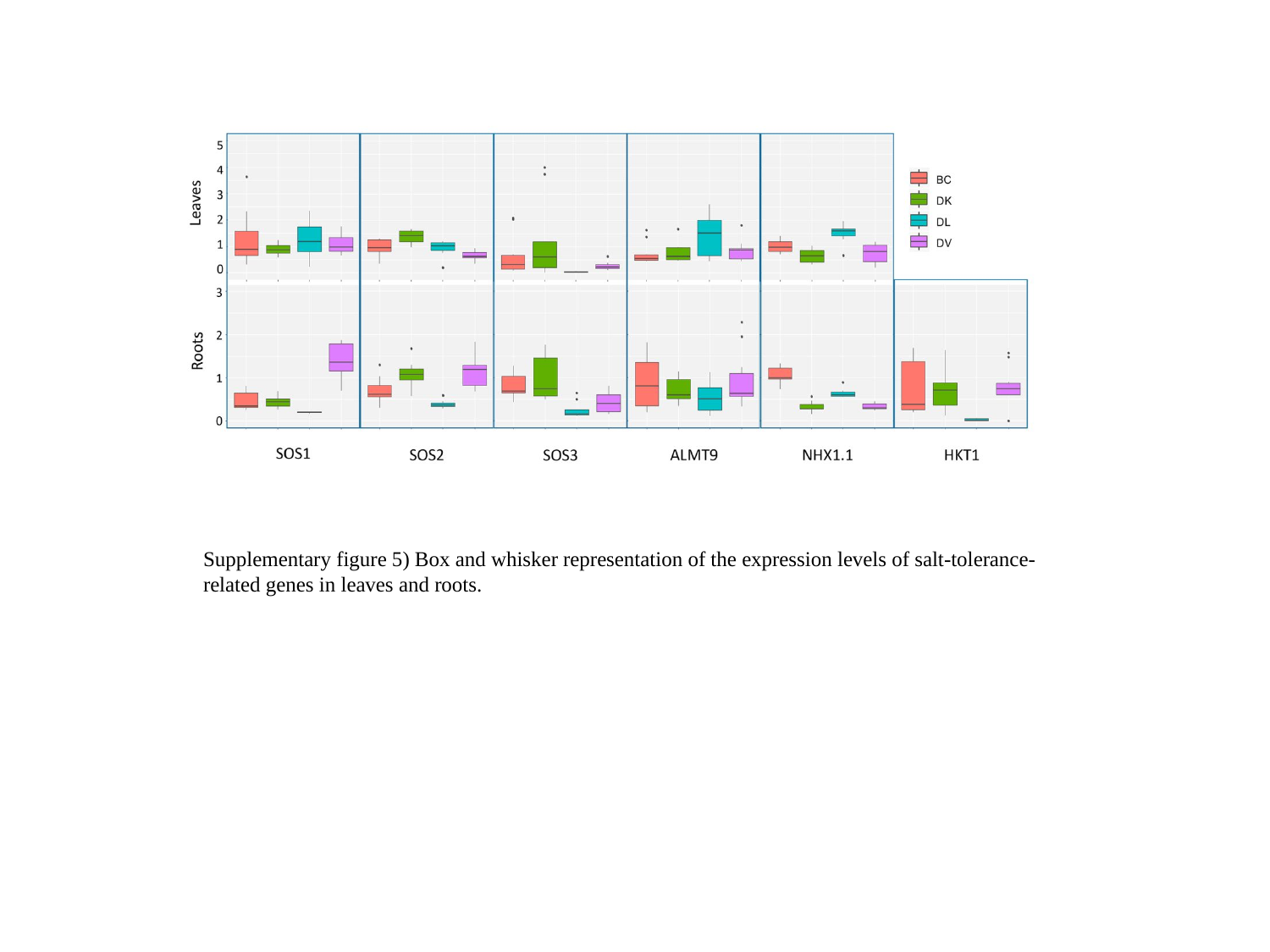

Supplementary figure 5) Box and whisker representation of the expression levels of salt-tolerance-related genes in leaves and roots.

## Slide 6
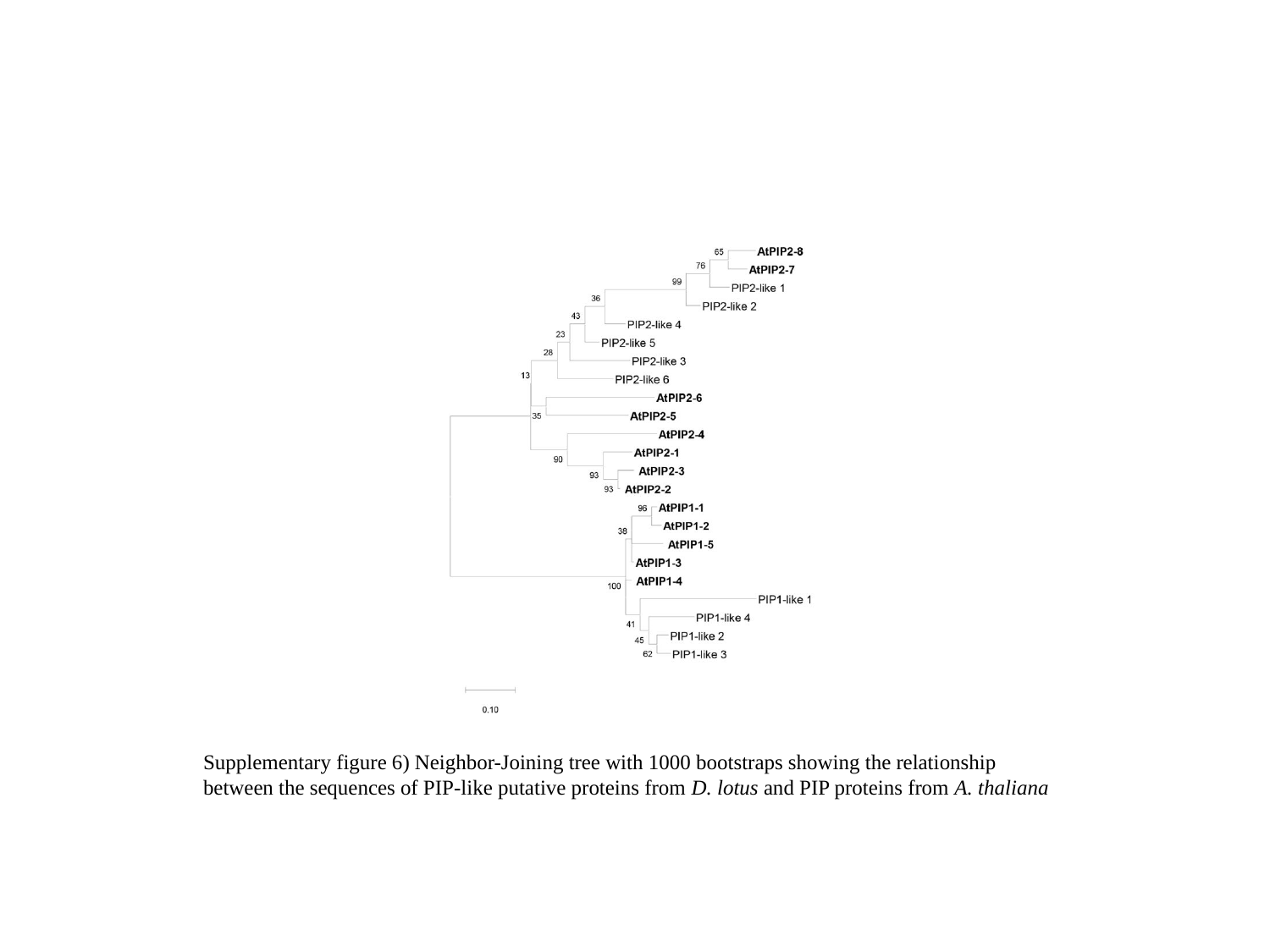

Supplementary figure 6) Neighbor-Joining tree with 1000 bootstraps showing the relationship between the sequences of PIP-like putative proteins from D. lotus and PIP proteins from A. thaliana
